# Skull Repair after Major Crush Injury

**DOI:** 10.1155/2011/749250

**Published:** 2011-10-05

**Authors:** H. Sudhoff, H. J. Hoff, M. Lehmann

**Affiliations:** ^1^Department of Otolaryngology, Head and Neck Surgery, Klinikum Bielefeld, Academic Teaching Hospital University of Münster, Münster University, Teutoburger Street 50, 33604 Bielefeld, Germany; ^2^Department of Neurosurgery, Evangelisches Krankenhaus Bielefeld, 33604 Bielefeld, Germany

## Abstract

Reconstructive surgery after trauma of the head and neck is a wide field in ENT surgery. The repair of bony defects often requires implantation of engineered prostheses. We present the case of a 48-year-old male patient who had suffered a major crush injury to his head resulting in a complex bony defect. A computer-assisted designed (CAD/CAM) Titanium implant was used for reconstruction. Direct prefabrication of the individually designed implant led to an excellent coverage of the bony defect and easy adaptation to the defect margins. *Results*. Treatment plan and surgery as well as implant design and manufacturing were performed in a multidisciplinary team. Skin expander implantation prior to reconstructive surgery ensured a tension-free closure. This team approach led to a satisfactory outcome for this patient. This case illustrates the necessity of a multidisciplinary approach for the optimum management of complex head and neck injuries.

## 1. Introduction

We illustrate our technique of skull repair using individually designed computer-aided titanium prostheses by an unusual case. The titanium prosthesis is individually CAD-designed using the DICOM high resolution CT dataset. This process is based on free-form surfaces and is carried out in several steps [1]: (1) derivation of the outer cranial contour from the unaffected neighboring contours; (2) determination of the implant thickness by projecting the outer contour more centrally as an inner contour; (3) determination of the implant margins by the borders of the defect for a precise and individual fit; (4) inclusion of fixation lugs, in which holes for titanium microscrews are drilled at the end of the CAD/CAM process. Based on this CAD model, the implant is manufactured in a numerically controlled milling machine (i.e., CAM) with an accuracy of less than 1 mm. The average implant thickness is 2-3 mm, and the implant body is provided with 8-mm holes homogeneously distributed over its surface for possible drainage beneath the implant and a more stable soft-tissue integration [2].

## 2. Case Report

**Figure 1:**
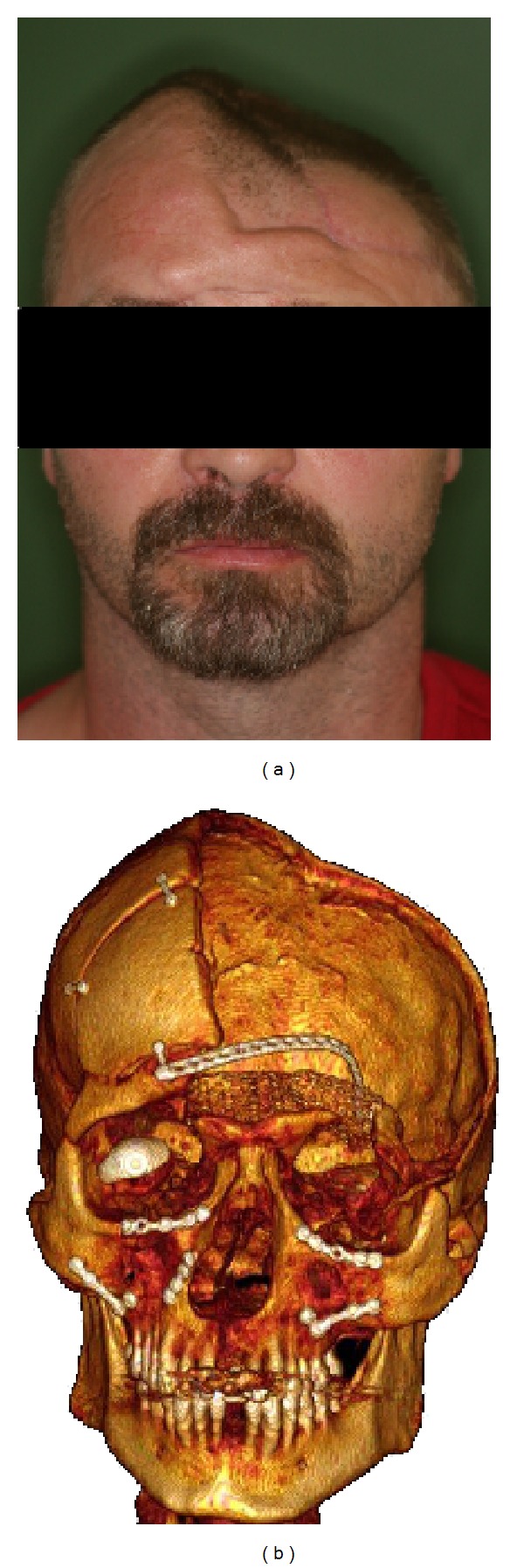
(a) Shows on the left an obvious skull deformity after crush injury. Bone is largely missing and the left orbital margin is severely compromised. The right eye is removed. (b) A composite 3D reconstruction of the patient's skull showing the extensive bone loss and sequelae of multiple skull and facial fractures.

A 48-year-old German safety officer suffered compression injury to his cranial vault whilst examining a pressing machine (Figure 1(a)). The accidental onset of the machine resulted in severe compression of his skull. This led to compound comminuted skull and facial fractures (parietal, sphenoid, frontal, orbital rim, ethmoid, maxillary, zygomatic, and nasal bones) (Figure 1(b)). This trauma left him with right-sided hemiplegia, right-sided total blindness, and loss of bony covering on the left side of his brain. Six months after primary trauma surgery, he presented to the ENT department for definitive reconstruction of the skull and right-sided dacryocystorhinostomy to treat his right-sided epiphora.

**Figure 2:**
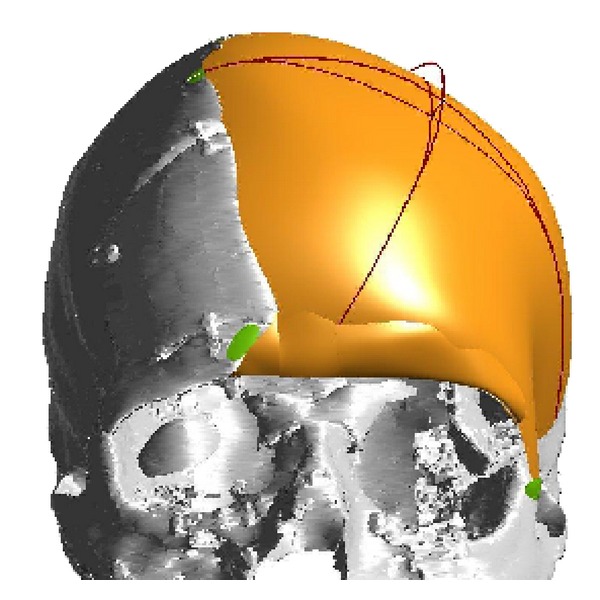
This figure shows a computerized reconstruction of the bony defect. This is used as a template for construction of a titanium cranioplasty prosthesis.

A computer-assisted designed/computer-assisted modeled (CAD/CAM) titanium implant for cranioplasty was manufactured using the DICOM high-resolution CT dataset (Figure 2). The patient underwent bilateral cranial tissue expander insertion 8 weeks prior to reconstructive surgery. Three months prior to reconstruction, a skin expander was implanted to ensure a tension-free closure of the defect.

**Figure 3:**
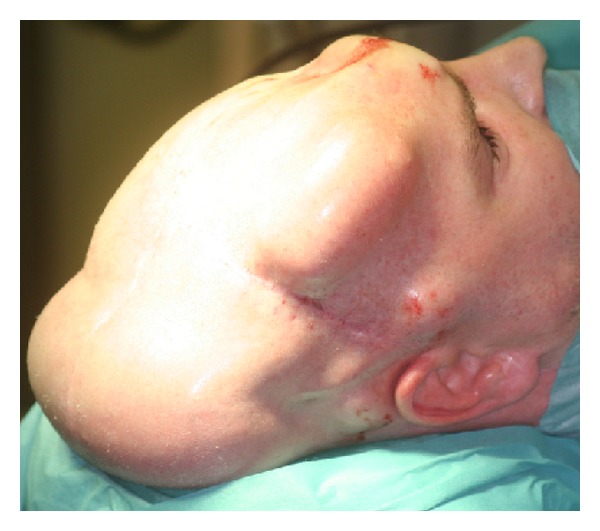
Prereconstruction photograph. Due to the expected skin loss, two inflatable skin expanders had been inserted.

**Figure 4:**
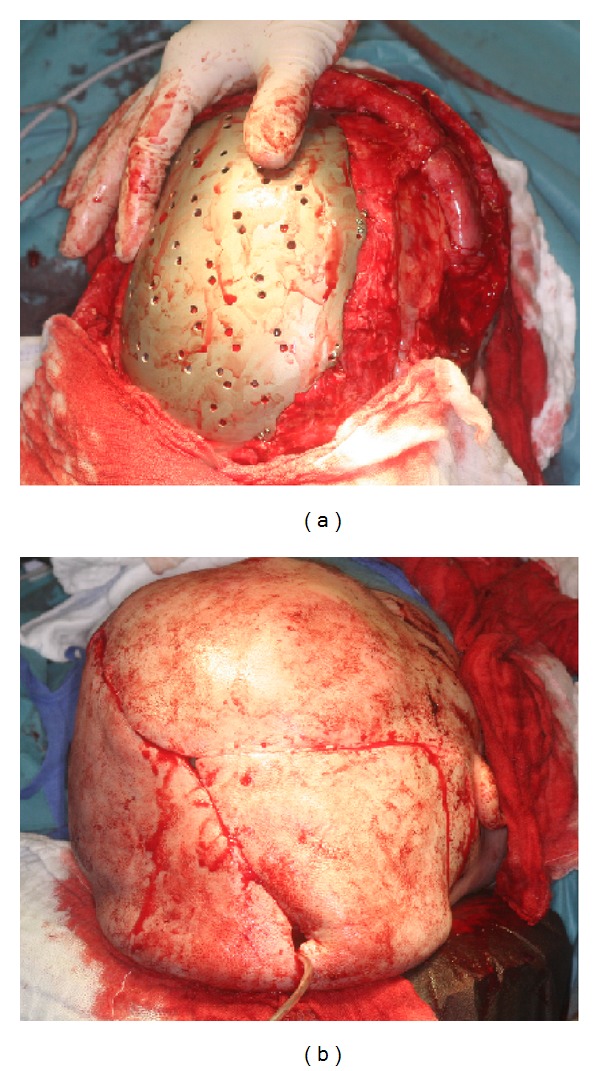
(a) Shows the “in situ” titanium cranioplasty. Note the fit and screw fixation to the defect margins. (b) Postoperative view after tension-free skin closure.

**Figure 5:**
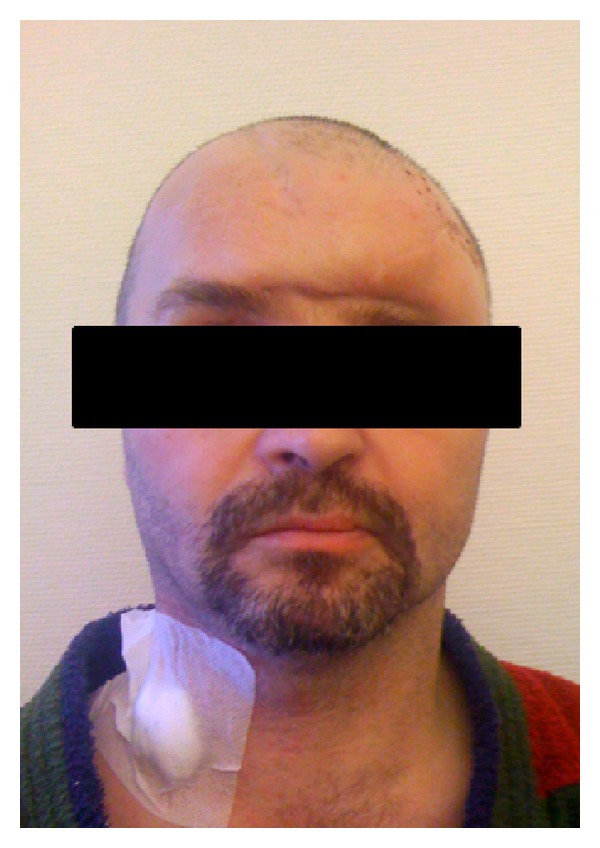
Showing the patient one week after surgery. The missing bone has been replaced by the titanium cranioplasty prosthesis. Wound healing was excellent and the excess skin flattened. A prosthesis will be fitted to replace his right eye.

After dissection of the overlying scared skin, the dura was exposed. The titanium cranioplasty prosthesis installed and fixed with self-drilling 7 mm screws (Figure 3). The fit was excellent. The previous tissue expansion resulted in tension-free skin closure over the implant (Figure 4(a)). Drains were placed for the initial 48 hours. Excess skin was left in expectation of flattening as healing occurred (Figure 4(b)). This was found to be the case at 1 week after surgery (Figure 5). The placeholder positioned during right-sided dacryocystorhinostomy was removed 3 months prior to the initial surgery.

## 3. Discussion

Reconstructive surgery after trauma of the head and neck is a wide field in ENT surgery. The repair of bony defects often requires implantation of engineered prostheses. Prefabricated titanium implants were found to be a valuable tool in reconstructive skull surgery and the material of choice for this severe skull defect resulting from compression injury [3, 4]. 

The stability and shock resistance of the CAD/CAM titanium plate is superior to that of the titanium mesh. Consequently, it is more useful in reconstructing large lesions of the skull, especially in those anatomical regions that are exposed to increased muscular traction forces from either side of the implant.

Skin expansion prior to skull reconstruction is very important since especially alloplastic implant material needs tension-free coverage with well-perfused skin.

Individual prefabricated CAD/CAM implants are technologically superior and clearly more expensive than their titanium mesh counterparts since they entail the complex and time-consuming process of data acquisition and transfer to the CAD/CAM facility, implant geometry design in an idealized technical fashion, and the computer-controlled milling out of the implant from a solid block of titanium—all in advance of the reconstructive procedure. Because of this cost- and time-related disadvantage, these implants may not be readily available and should be reserved to selected cases [4].

The application computer-assisted designed/computer-assisted modeled (CAD/CAM) titanium implants for cranioplasty is a surgical strategy for extreme cases but also illustrates an elaborate interdisciplinary treatment in computer-assisted surgery (CAS). Our case highlights the necessity of a multidisciplinary team approach for the optimum management of complex head injuries.
